# Hybrid model of the context dependent vestibulo-ocular reflex: implications for vergence-version interactions

**DOI:** 10.3389/fncom.2015.00006

**Published:** 2015-02-09

**Authors:** Mina Ranjbaran, Henrietta L. Galiana

**Affiliations:** Department of Biomedical Engineering, McGill UniversityMontreal, QC, Canada

**Keywords:** sensory-motor mapping, vestibulo-ocular reflex, context dependent reflex, mathematical model, disconjugate eye movement, ocular nystagmus

## Abstract

The vestibulo-ocular reflex (VOR) is an involuntary eye movement evoked by head movements. It is also influenced by viewing distance. This paper presents a hybrid nonlinear bilateral model for the horizontal angular vestibulo-ocular reflex (AVOR) in the dark. The model is based on known interconnections between saccadic burst circuits in the brainstem and ocular premotor areas in the vestibular nuclei during fast and slow phase intervals of nystagmus. We implemented a viable switching strategy for the timing of nystagmus events to allow emulation of real nystagmus data. The performance of the hybrid model is evaluated with simulations, and results are consistent with experimental observations. The hybrid model replicates realistic AVOR nystagmus patterns during sinusoidal or step head rotations in the dark and during interactions with vergence, e.g., fixation distance. By simply assigning proper nonlinear neural computations at the premotor level, the model replicates all reported experimental observations. This work sheds light on potential underlying neural mechanisms driving the context dependent AVOR and explains contradictory results in the literature. Moreover, context-dependent behaviors in more complex motor systems could also rely on local nonlinear neural computations.

## 1. Introduction

The vestibulo-ocular reflex is an involuntary eye movement that stabilizes gaze in space during head movements for clear and blur-free vision. The rather simple neural substrate of the VOR, the so-called *three neuron arc* (de No, 1933), makes it an appropriate model to study sensory-motor behavior. Rotational and translational head movements are sensed by the vestibular system (semicircular canals and the otolith organs) in the inner ear. Vestibular afferents relay sensory information to the vestibular nuclei (VN) and prepositus hypoglossi (PH) centers in the brainstem. These centers act as the main controller and combine sensory signals with internal efference copies of the controlled plant(s), eye orientation, to drive motor-neurons appropriately. Extraocular muscles then apply torques on the eyeball that result in the eye movements.

**Figure 1:**
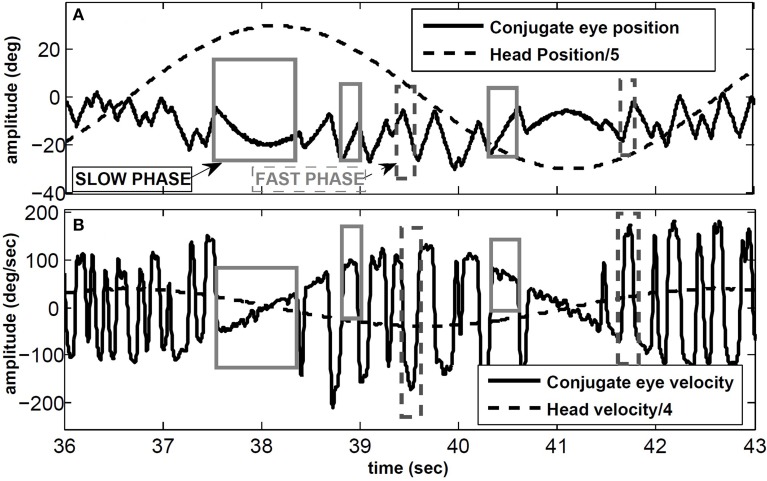
**VOR in response to sinusoidal head rotation recorded with EOG**. **(A)** Conjugate eye position and scaled head position (degree). **(B)** Conjugate eye velocity and scaled head velocity (degree/s). Sample slow and fast phase segments are marked with gray and dashed-gray rectangles, respectively.

VOR nystagmus consists of compensatory (slow phase) and reorienting (fast phase) segments. The slow phases of the VOR stabilize gaze in space by moving the eyes in the opposite direction to the head movement, while the fast phases redirect the gaze at high speeds in the direction of the head velocity. We focus on the angular VOR (AVOR), tested with passive whole-body rotation in the dark while recording conjugate (eye movements in the same direction) or monocular horizontal eye movements. Figure 1 shows an example of the VOR during sinusoidal head rotations using electrooculography (EOG) in the dark: some slow and fast phase segments are marked. The sawtooth-like pattern of the eye movement is a characteristic of most types of eye movements and is known as ocular nystagmus. In clinical tests, the VOR is characterized by its *gain* defined as the ratio of peak eye velocity to peak head velocity during harmonic testing or short pulse perturbations.

While the head movements initiate the VOR, this reflex is also influenced by contextual factors such as viewing distance (Viirre et al., 1986; Crane and Demer, 1998). Since the eyes are not centered on the head, holding gaze on a near target requires more ocular rotation than for a relatively far target during head movements. In other words, the AVOR gain increases as a function of decreasing fixation distance, that can be described geometrically. The majority of models that attempted to explain target-distance dependent VOR responses relate this property to (i) an internal signal proportional to the inverse of target distance that scales VOR gain (Viirre et al., 1986; Chen-Huang and McCrea, 1999), (ii) cortical computations (Snyder and King, 1992), (iii) parametric changes (Green, 2000), (iv) multiplication of vestibular and eye position signals (Zhou et al., 2007) or (v) parallel linear-nonlinear pathways (Lasker et al., 1999). All these models are only focused on the slow phases of VOR nystagmus.

**Figure 2:**
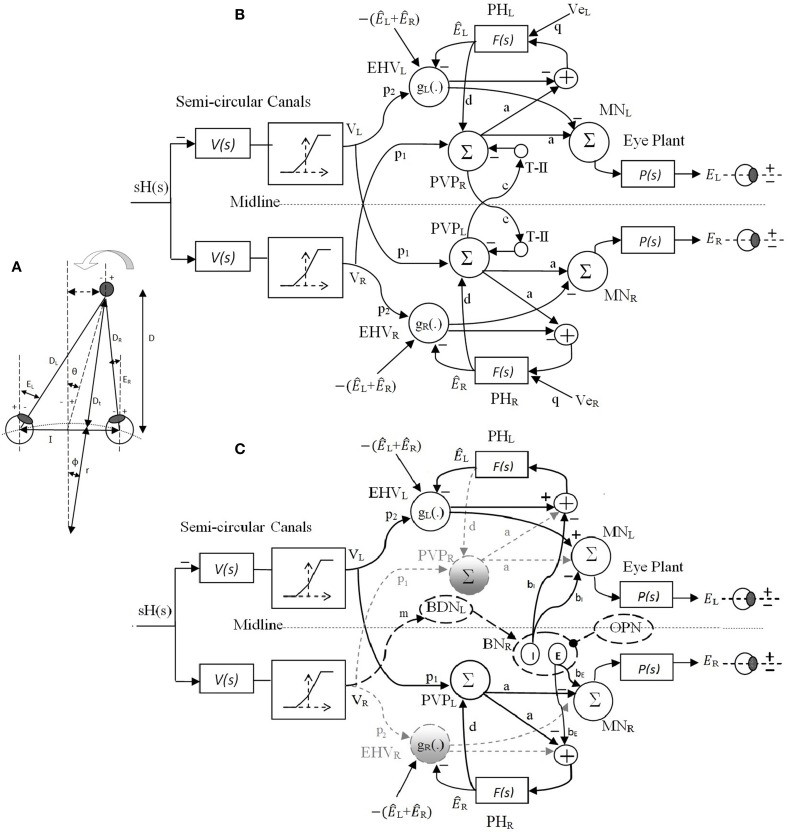
**(A)** Right and left eye positions (*E_R_*, *E_L_*) for an eccentric target at a location given by (*D*, θ) during head rotation about radius *r*. *I* is the interocular distance. **(B)** Bilateral model of slow phase horizontal AVOR in the dark. **(C)** Model structure for a rightward fast phase. Inactive projections and paused cells are indicated by dashed-gray lines. Long dashed-black lines are the centers that are only active during fast phase and solid black lines are shared projections during both slow phase and rightward fast phase of the VOR. Silenced cross midline projections are not shown for simplicity. PVP cells are located on the opposite side from their physical location to better view the connections crossing the midline. See Table 1 for projection weights that are shown on the connections. Projection weights that are not shown are assumed to be 1. Parts **(A)** and **(B)** partially adapted from Ranjbaran and Galiana (2013a).

In our recent work (Ranjbaran and Galiana, 2013a), we presented a nonlinear bilateral model for AVOR slow phases in the dark. The model is developed based on known realistic physiological mechanisms and anatomical connections including the semicircular canals, the VN and PH neural populations, motor-neurons and eye plants (Figure 2A). Based on geometrical relations, we showed that combining monocular and vergence angle (eye movements in opposite directions) information is sufficient to locate a target in space relative to the eyes. By assigning properly tuned nonlinear neural computations at the VN level, this slow phase model is capable of replicating target-distance dependent VOR responses that meet geometrical requirements. Nonlinear computation in neural responses, so-called *gain modulation*, exists in many cortical and subcortical areas (Salinas and Sejnowski, 2001). Different mechanisms are proposed to explain them, such as recurrent neural networks (Salinas and Abbott, 1996), changes in the synchrony of inputs to a neuron (Salinas and Sejnowski, 2000) or varying the level of background synaptic input (Chance et al., 2002). In this slow phase model (Ranjbaran and Galiana, 2013a), it is postulated that the sensitivity of the VN cells to vestibular signals modulates nonlinearly with eye position and vergence state, enabling auto-adjustment of the VOR to the set point of both eyes- a great improvement over the initially proposed model that only used ipsilateral monocular signals (Khojasteh and Galiana, 2009b). In addition to the near ideal AVOR gain modulation with target distance, the central premotor responses in that model are also consistent with experimental observations. The model also reproduces experimental observations of the VOR responses with simulated unilateral canal plugging, an emerging property. Due to nonlinearities in the sensors and premotor circuits, the model predicted a disconjugate VOR in the dark. However, prior explorations of the model behavior were examined only during high frequency head pulses or low amplitude sinusoidal rotations to remain in the range of feasible eye rotations. In order to have more relevance to the clinical VOR, we now examine the predicted responses to low frequency sinusoidal and large head rotations. This requires the implementation of a fast phase circuit to replicate realistic nystagmus patterns in the AVOR and compare simulations to experimental data.

Classically, the two phases of the VOR are believed to be generated by independent and parallel pathways as originally suggested by Chun and Robinson (1978). Based on this approach, the two phases function independently from each other and a switching strategy implements the timing of changes from one system to the other. Such a black box approach was also employed by other researchers to study VOR slow and fast phase interactions (Winters et al., 1984). However, more recent data demonstrate that slow and fast phases of the VOR share efference copies of eye position from PH and premotor cells in VN (Fukushima and Kaneko, 1995). The first model to include shared connections between the slow and fast circuit was proposed by Galiana (1991) where distinct dynamics for slow and fast phases are generated here through structural modulation. In other words some of the projections during slow phases alter their response characteristics during a fast phase: e.g., position vestibular pause (PVP) and eye head velocity (EHV) cells in the VN pause for ipsilaterally directed fast-phases (McFarland and Fuchs, 1992) and burster cells that are only active during fast phases or saccades, play an important role in facilitating response changes on premotor cells (Kitama et al., 1995). It should be noted that structural modulation does not refer to any change in anatomical connectivity, but rather to changes in the available set of active pathways (Galiana, 1991). Another comparable physiologically relevant model was also developed using realistic spiking neurons that replicated VOR nystagmus in the guinea pig with shared connections between the slow and fast circuits (Cartwright et al., 2003). However, both these models do not address VOR gain modulation with target distance nor vergence interactions. The hybrid model developed by Khojasteh and Galiana (2009b) considered VOR gain modulation; however, they considered a nonlinear block in a feedback loop in their slow phase model to account for VOR gain modulation which resulted in variable VOR dynamics with context and head velocity profiles. Moreover, in their model only ipsilateral monocular signals are used to modulate VOR gain, thus it is not possible to test VOR gain modulation during simultaneous vergence goals and harmonic vestibular inputs.

In this paper, the fast phase circuit shares premotor centers with the bilateral nonlinear slow phase circuit previously presented (Ranjbaran and Galiana, 2013a) to form the VOR hybrid model. A novel feature is that for the first time, VOR and vergence interaction is included in a physiologically relevant hybrid model that can replicate experimental observations, i.e., modulation of the VOR gain in response to simultaneous variable vergence goals and vestibular inputs in the dark. A viable switching strategy is also implemented to trigger and stop VOR fast/slow phases, originally suggested by Galiana (1991). Simulation results are presented to evaluate the performance of this hybrid model under different rotation profiles and the results are compared with experimental observations. Such a model allows new interpretations of the underlying mechanisms in the VOR system and explains contradictory observations in experiments. Preliminary results are presented in Ranjbaran and Galiana (2013b).

The remainder of this paper is organized as follows. Materials and Methods in Sections 2.1 and 2.2 review briefly the reference coordinates and the previously developed slow phase model. Sections 2.3 and 2.4 describe the fast phase model and the nystagmus strategy. Simulation results in Section 3 are followed by discussion and concluding remarks in Section 4.

## 2. Materials and methods

In developing mathematical representations for the slow and fast phases of the VOR, we are modeling population responses of cells. Therefore, each element of the models represents the average behavior of a particular cell type rather than the response of any individual cell. Moreover, only firing modulation around a population resting rate is considered; biases due to resting rates are not included and a negative firing rate refers to a cell firing below its resting rate. Finally, we wish to represent the simplest model that can replicate general VOR characteristics, i.e., a minimalist approach. Adding more projections and loops between elements in a bilateral model will only affect the current assigned projection weights and not the general characteristics of the model.

### 2.1. Reference coordinates

For each eye, zero position is defined as looking straight ahead at optical infinity; temporal deviations are considered positive and nasal deviations, negative. Conjugate and vergence eye positions are thus defined as $E_{conj}=\frac{1}{2}(E_{R}-E_{L})$ and *E_verg_* = −(*E_R_* + *E_L_*), where *E_R_* and *E_L_* refer to the right and left eye angle, respectively (see Figure 2A).

### 2.2. Slow phase model

The original nonlinear model for slow phases of the AVOR (Figure 2B) is described in detail in Ranjbaran and Galiana (2013a). The input is head velocity, *sH*(*s*), sensed by semicircular canals. The canals are modeled as high-pass filters of head velocity, $V(s)=\frac{sT_{c}}{sT_{c}+1}$, followed by a static nonlinearity on sensory modulation. The nonlinear block accounts for the mechano-neural transduction process that causes asymmetric changes in the firing rate on the primary afferents (Goldberg and Fernandez, 1971). The nonlinear block has asymmetric gains around zero (*k_negative_* = 0.4 and *k_positive_* = 0.6) and limits the primary afferent output *V_R,L_* by saturation (+110 spikes/s) and cutoff levels (−90 spikes/s), appropriate for primary vestibular afferents with the 90 spikes/s resting rate. PVP and EHV cell populations in the VN are distinct in the model and receive sensory projections from the canals as well as efferent copies of eye position from PH. T-II in our model refers to type II neurons in the medial VN (Shimazu and Precht, 1966) that receive projections from the contralateral VN. We assume that contralateral VN projections arise from from PVP cells and form a feedback loop between the two sides of the VN (Keller and Precht, 1979). These commissural pathways play an important role in the dynamics of the VOR system (Galiana and Outerbridge, 1984). Premotor PVP and EHV cells project to motor neurons (MN) to drive the eye plants. The eye globe and muscles smooth the motoneural signals. This is represented mathematically as a low-pass transfer function. Therefore, the eye plants as well as neural filters in PH are modeled with first order low pass dynamics as $P(s)=\frac{k_{p}}{sT+1}$ and $F(s)=\frac{k_{f}}{sT+1}$. Here, we assume that efference copies of the ipsilateral monocular eye position, *Ê*_*R*,*L*_, and of the vergence eye position, *Ê*_*verg*_, reach EHV cells and define their sensitivities (gain) to vestibular signals in a nonlinear fashion; i.e., *EHV*_*R,L*_ = *g*_*R,L*_{*Ê*_*R,L*_, *Ê*_*verg*_} *p*_2_ *V*_*R,L*_, where *g_R,L_*{.} is the nonlinear sensitivity of EHV cells to vestibular afferents. These nonlinear computations account for the target distance related gain modulation of the VOR.

The equations for conjugate and vergence angles in the model are

(1a) $$E_{conj}=\frac{k_{p}(c-1)(g_{L}\left\{.\right\}p_{2}V_{L}-g_{R}\left\{.\right\}p_{2}V_{R})-ak_{p}p_{1}(V_{L}-V_{R})}{2\left((c-1)(Ts+1)+adk_{f}\right)}$$

(1b) $$E_{verg}=\frac{k_{p}(c+1)(g_{L}\left\{.\right\}p_{2}V_{L}+g_{R}\left\{.\right\}p_{2}V_{R})-ak_{p}p_{1}(V_{L}+V_{R})}{(c+1)(Ts+1)-adk_{f}}$$

Modulation of *g_R_*(.) and *g_L_*(.) changes the context gain but not the system dynamics (poles). In simpler terms, the sensory signals from the semi-circular canals are smoothed and tuned by the brainstem circuits to generate the eye movement, described by nonlinear low pass dynamics. For complete justification of the model elements and connections see (Ranjbaran and Galiana, 2013a).

The slow phase model is originally designed to replicate VOR responses in the dark with no visual cue. In order to evaluate the effect of far vs. near target flashes during sinusoidal rotation in the dark, additional inputs to trigger vergence eye movements are required. It is postulated that viewing a flashed target causes signals to be relayed to the neural filters in the PH from any cortical or brainstem center coding visuomotor error commands, such as superior colliculus (SC) (Cova and Galiana, 1996; Green, 2000). We define these visual error signals (*Ve_R_* and *Ve_L_*) as additional input signals to the PH (Figure 2B). In the absence of head velocity input, i.e., *V_R,L_* = 0, the conjugate and vergence response to *Ve_R,L_* are obtained as

(2a) $$E_{conj}=\frac{ak_{p}k_{f}dq(Ve_{R}-Ve_{L})}{2(Ts+1)\left((c-1)(Ts+1)+adk_{f}\right)}$$

(2b) $$E_{verg}=\frac{-ak_{p}k_{f}dq(Ve_{R}+Ve_{L})}{(Ts+1)(c+1)(Ts+1)-adk_{f}}$$

Assigning identical visuomotor error commands, i.e., *Ve_R_* = *Ve_L_*, results in pure vergence with no conjugate response due to the bilateral structure of the model. It should be noted that we are not including light conditions or continuously visible targets in the dark since the VOR dynamics will change as additional visual loops are added to the circuit (Green, 2000). This is a question for future studies.

Two sets of parameters are provided in Table 1 that simulate two different time constants for the conjugate slow phase system obtained from Equation (1A), i.e., $T_{conj}=\frac{T(c-1)}{adk_{f}+c-1}$ equals 5 s or 1.2 s and $T_{verg}=\frac{T(c+1)}{-adk_{f}+c+1}$ from Equation (1B) equals 0.4 s with both parameter sets. By simply changing projection weights or filter gains, e.g., *c* and *d* or *k_f_*, new time constants for the model can be obtained. However this then requires retuning of the nonlinear surfaces at the EHVs. The canal time constant is set to *T_c_* = 6 s. Nonlinear surfaces assigned to the EHV cells (Ranjbaran and Galiana, 2013a) are provided in Appendix 5.1. Here interocular distance is: *I* = 6 cm and the axis of rotation is: *r* = *r_head_* = 8.8 cm.

**Table 1:** **Numerical values of the model parameters**.

***T_conj_***	***T_verg_***	***p_1_***	***p_2_***	***c***	***a***	***d***	***m***	***b_I_***	***b_E_***	***k_f_***
5 s	0.4 s	1	0.5	0.58	0.75	0.65	1	10	10	0.813
1.2 s	0.4 s	1	0.5	0.5	0.75	0.77	1	10	10	0.65
***T_conj_***	***T_verg_***	***k_p_***	***k_ff_***	***k_pf_***	α	***q***	***T***	***T_c_***	***ON-Th***	***OFF-Th***
5 s	0.4 s	0.407	0.3	0.15	0.2	0.135	0.3	6	90	−5
1.2s	0.4s	0.325	0.3	0.15	0.2	0.1	0.3	6	60	−5

### 2.3. Fast phase model

The model structure for a rightward fast phase circuit is shown in Figure 2C. A leftward fast phase is generated with a mirror image of this model. Similar to the slow phase circuit, only modulations in cell populations are provided. Summing junctions are linear except for the nonlinear EHV cells (Ranjbaran and Galiana, 2013a). The bilateral structure of the slow phase system with reciprocal signals across the midline switches to a unilateral structure during fast phases. This is the result of silenced VN cells such as the ipsilateral PVPs and EHVs as well as the cross-midline projections during a fast phase (gray dashed lines and circles in Figure 1B, cross midline projections are not shown for simplicity). Omnipause neurons (OPN) and burster-driving neurons (BDN) as well as excitatory and inhibitory burst neurons (EBN and IBN) are included in the fast phase circuit (long dashed black lines and circles in Figure 1). OPNs are located near the midline of the pons and act as triggers for the initiation of fast eye movements in all directions (Scudder et al., 2002). OPNs discharge at high firing rate and exert a tonic inhibition on premotor BNs during slow eye movements and fixation. Prior to a fast eye movement or saccade, OPNs cease firing, remain silent during the saccade, and resume firing as the saccade ends (Yoshida et al., 1999). OPNs receive projections from the SC as well as projections from cells in the medial VN (Ito et al., 1986). In our model, it is assumed that the projections from the medial VN, specifically PVPs, to the OPNs play a role in triggering and ending the fast phases (see Section 2.4). BDNs are located below the PH and are found to be excited by contralateral horizontal head rotation and they send projections to contralateral BNs (Kitama et al., 1995). We assume BDN excitation is a result of an excitatory vestibular drive that comes from contralateral vestibular-only (VO) cells. BDNs also modulate with a PH response (eye position efference copies) to close the loop and shape bursts during fast phases (Kitama et al., 1995). Therefore, the output signal from left BDNs during rightward fast phase is: *BDN_L_* = *m* × *V_R_* − α × *Ê*_*R*_ where α is the projection weight from the PH to contralateral BDN (connections are simplified in Figure 1). BDNs project to contralateral EBNs and IBNs that are located in the reticular formation and have monosynaptic connections to abducens motoneurons. EBNs send excitatory projections to ipsilateral MNs while IBNs with similar firing patterns send inhibitory projections to contralateral MNs. In our model, the same projections to MNs are sent to PH neurons that produce efferent copies of eye position for VN cells (Fukushima and Kaneko, 1995).

In order to achieve faster dynamics in the fast phase circuit, it is assumed that the feedback loops including PVP and EHV cells between the VN and PH nuclei change their net sensitivity direction as originally suggested in Galiana (1991). This can be a result of competition between parallel inhibitory and excitatory projections from PVP and EHV cells whose balance is modified by EBN/IBN effects. Projections from burst neurons to VN are studied in Igusa et al. (1980) and have been used in former models and studies (Curthoys, 2002; Cartwright et al., 2003). We assume that the population response of the VN cells is a mixed combination of individual inhibitory and excitatory projections. During slow phases, the population response is dominated by the excitatory PVPs and inhibitory EHVs as the burst neurons are silenced. During a fast phase, however, as the burst neurons become dis-inhibited, along with silencing of the ipsilateral PVPs and EHVs, the inhibitory projections dominate the response of PVPs and excitatory projections dominate the response of EHVs; thus, the net projections from contralateral PVPs and EHV populations appear to change their direction of sensitivity. Similar incrementing-decrementing behavior is also observed on the BDN activity during slow-fast intervals (Ohki et al., 1988). The change in effective connectivity assumed here does not conflict with basic knowledge about the neural firing patterns. Thus, in our model during a fast phase, PVPs and EHVs contralateral to the fast phase direction change their sensitivity and their activity profiles decay.

As in the slow phase model, the eye plants and neural filters in the PH remain as first-order low-pass dynamics as $P_{f}(s)=\frac{k_{pf}}{Ts+1}$ and $F_{f}(s)=\frac{k_{ff}}{Ts+1}$. EHV cells are silenced during ipsilateral fast phases and are active during contralateral fast phases with the same nonlinear sensitivity to the ipsilateral canal signal, i.e., *g*(.) (Ranjbaran and Galiana, 2013a). Due to the unilateral structure of the fast phase of the VOR, distinct monocular dynamics for eye responses are obtained during fast phases, i.e., during a rightward fast phase (see Appendix 5.2)

(3a) $$E_{R}=\frac{k_{pf}(-ap_{1}V_{L}+mb_{E}V_{R})}{Ts+1+k_{ff}(ad+b_{E}\alpha )}$$

(3b) $$E_{L}=\frac{k_{pf}\left(\beta_{1}V_{L}+\beta_{2}V_{R}\right)}{\left(1+Ts)(Ts+1+k_{ff}(ad+b_{E}\alpha )\right)}$$

where

(4) $$\{\begin{array}{l} \beta_{1}=(1+Ts+k_{ff}(ad+b_{E}\alpha ))g_{L}\left\{.\right\}p_{2}-b_{I}\alpha k_{ff}ap_{1}\\ \beta_{2}=-(1+Ts+k_{ff}(ad+b_{E}\alpha ))mb_{I}+b_{I}\alpha k_{ff}mb_{E} \end{array}$$

During a leftward fast phase, the dynamic equations for *E_R_* and *E_L_* are obtained by switching the *R* and *L* subscripts in Equations (3A,B). The above equations imply that monocular eye trajectories have different dynamics during rightward and leftward fast eye movements.

The model parameters (Table 1) are selected to preserve the stability of the fast phase system with a small time constant.

### 2.4. Strategy for nystagmus

So far, the slow and fast phase models with shared connections are described. However, an important feature of the VOR is the switching mechanism between these two phases. The linear range of the VOR is improved by nystagmus as eye excursions are kept inside a reasonable limit. Therefore, a proposed switching strategy is based on limiting eye deviations by avoiding cut-off and saturation limits in the responses of premotor neurons (Galiana, 1991). It is known that the activity of OPNs acts as a logical circuit to trigger and end a fast phase. In this model, the OPN circuit constantly monitors the output of PVPs. If the firing rate of PVPs on one side reaches a threshold (ON-Th spikes/s), a fast phase is triggered ipsilateral to the PVPs' side. During the fast phase, the ipsilateral PVPs and EHVs are silenced and the contralateral PVPs and EHVs change direction and decay as explained in the fast phase circuit structural modulation. The fast phase ends as the firing rate of the contralateral PVPs decays to a second threshold (OFF-Th spikes/s). The fast phase intervals are therefore generated in the same direction as head movement and eye position signals are kept below their physical limits. ON-Th and OFF-Th control the frequency of fast phases and their duration. For instance, with fixed model parameters and dynamics, increasing ON-Th results in later triggering of fast phases and lowering the OFF-Th leads to longer fast intervals. We have imposed a refractory period of 20 ms in the model after switching back to a slow phase to enforce a minimum time interval before triggering a new fast phase.

The performance of the model under different conditions are provided next. All simulations were performed using MATLAB Simulink (The MathWorks Inc., USA), with a first order Euler approximation and a step size of 1 ms.

## 3. Results

The model is designed to simulate the human AVOR responses during yaw rotations around a vertical axis centered on the head. We focus here on the global, behavioral aspects of the AVOR model rather than on individual components. PVP and EHV firing behavior is previously addressed in Ranjbaran and Galiana (2013a).

### 3.1. Response to sinusoidal rotation in darkness

**Figure 3:**
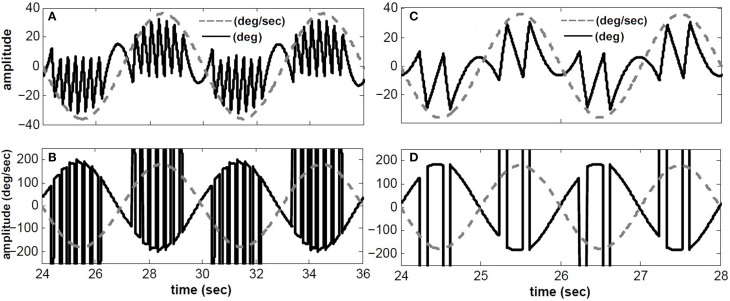
**Simulated conjugate eye position (top) and conjugate velocity (bottom) in response to sinusoidal head velocity rotation (amplitude = 180 degree/s)**. **(A,B)** input frequency is 1/6 Hz. **(C,D)** input frequency is 1/2 Hz. *solid-black* → top: conjugate position(degree)- bottom: conjugate eye velocity (degree/s), *dashed-gray* → top: head velocity/5 (degree/s)- bottom: head velocity (degree/s).

Figure 3 depicts the response of the hybrid model with *T_conj_* = 5 s at two different rotation frequencies: 1/6 Hz (A,B) and 1/2 Hz (C,D) with velocity peaks of 180 degree/s. As in experimental observations, the number of fast phases *per cycle* decreases for higher frequency sinusoidal head rotations. In other words, fast phases are triggered more often during low frequency head rotations. This is due to the band pass characteristics of the central neurons in the VOR pathway. At lower frequencies the gain of central neurons is higher which increases the possibility of exceeding their firing thresholds and triggering a fast phase. Furthermore, at a given rotation frequency, fast phases are more frequent at the higher head velocity levels; consistent with experimental observations (Buettner et al., 1978).

**Figure 4:**
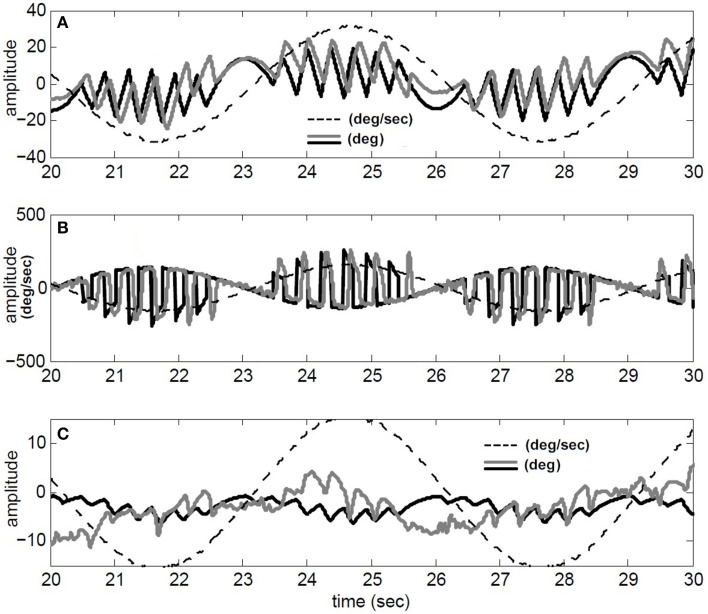
**Simulation results compared to recorded VOR nystagmus**. **(A)** Conjugate eye position (degree) and scaled (1/5) head velocity (degree/s). **(B)** Conjugate eye velocity (degree/s) and head velocity (degree/s) **(C)** Vergence eye position (degree) and scaled (1/5) head velocity (degree/s). black → Simulated, gray→ Recorded, dashed-gray→ Head velocity.

We have also compared our model performance with *T_conj_* = 1.2 s in response to a specific rotation profile (180 degree/s at 1/6 Hz) where binocular records are available in our archive; for details of the experiment see (Khojasteh and Galiana, 2009a). Figure 4 depicts the recorded conjugate eye position (A) and eye velocity (B) (gray), as well as our model responses to this rotation stimulus (black). Clearly, general nystagmus characteristics in the simulation and data are similar: the amplitude of conjugate eye position, the number of fast phases and their timing, suggest that the switching mechanism is plausible.

Given nonlinear canals and nonlinear premotor (EHV) computations, eye movements are disconjugate and a vergence component is now present in the response of our hybrid model to head perturbations. This vergence response shows a carrier frequency that is twice that of the stimulus, as also seen in the experimental data (Figure 4C). The peak-to-peak amplitude of the vergence component is greater than EOG resolution limits, suggesting that it cannot be a result of inappropriate calibration in the binocular recording. Instead, as predicted from the nonlinear model, this vergence component can be a direct result of nonlinearities at the premotor and sensory levels. It should be noted that here we did not attempt to identify a system directly from eye recordings but rather compare the general characteristics of recorded AVOR and our model response.

### 3.2. Context dependent response to sinusoidal rotation

The experimental work of Paige et al. (1998) studied the role of fixation distance in adjusting the gain of the VOR during *sinusoidal* angular head rotation. Here, we will test the response of our model during sinusoidal rotations while fixating on a near or far flashed target. Vergence movement as a result of fixating a target in the model is obtained by assigning proper visuomotor error commands, i.e., *Ve_R,L_*.

Starting with zero initial conditions (i.e., looking straight ahead at optical infinity), *E_R_*(0) = *E_L_*(0) = 0, *Ve_R_* = *Ve_L_* are set to replicate a flashed target in the dark appearing in the sagittal plane between the eyes. This flashed target appears 5 s after the start of head rotation, at *D* = 43 cm from the eyes requiring 8 degree of vergence (given the interocular distance of *I* = 6 cm; see Ranjbaran and Galiana, 2013a for geometrical relations). At *t* = 10 s, a new flashed target appears at *D* = 21 cm that requires 16 degree vergence. At *t* = 15 s a far flashed target appears to reset vergence to zero.

**Figure 5:**
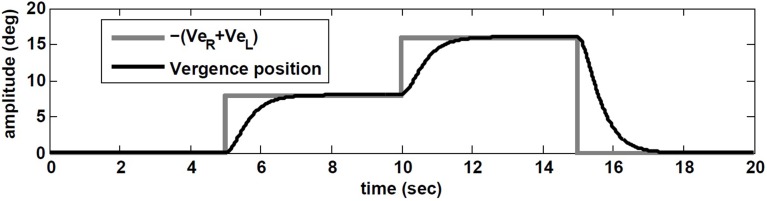
**Vergence eye movement in response to visuomotor command (*Ve_R,L_*) with stationary head, while orienting to flashed target at different distances**.

**Figure 6:**
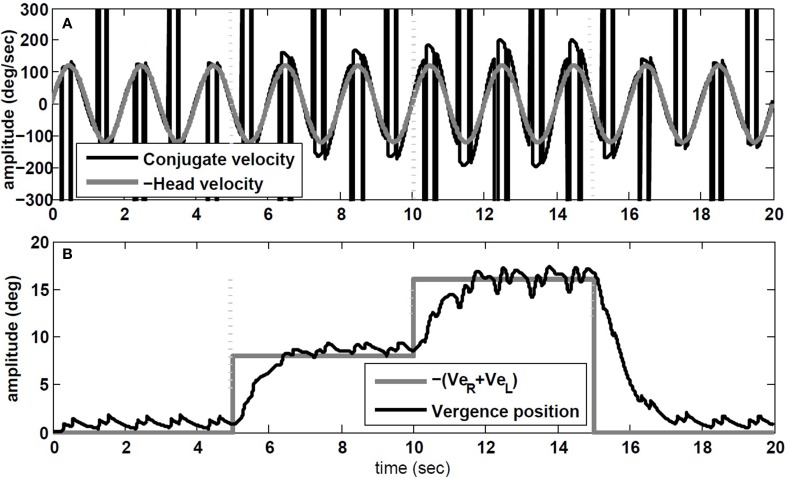
**Simulation results in response to head rotation (0.5 Hz, 120 degree/s) and concurrent visuomotor input. (A)** Conjugate eye velocity (degree/s) and negative head velocity (degree/s). **(B)** Vergence eye position (degree) and visuomotor command (*Ve_R_* + *Ve_L_*, gray). Vertical dashed lines mark the time of change in the visuomotor command.

The simulation results are obtained with model parameters where *T_conj_* = 1.2 s and *T_verg_* = 0.4 s (see Table 1). In the absence of head rotation, there is no conjugate eye movement, only a vergence response (Figure 5). During sinusoidal head rotation at 0.5 Hz with 120 degree/s peak velocity and the same visuomotor inputs, the conjugate and vergence AVOR responses are appropriate (Figure 6). The gain of the VOR (peak of envelope of eye velocity/peak head velocity) is near unity during the first 5 s with no visuomotor response as expected. During *t* = 5 → 10 s interval, the first flashed target, causes a VOR gain increase from unity to compensate for the visuomotor command and the vergence movement. As the second target appears at *t* = 10 s, a still larger vergence is required and therefore the VOR gain also increases further. As the final far target appears at *t* = 15 s, the vergence position decays to zero (looking far ahead) and the AVOR gain decreases smoothly to default unity. This is in agreement with the experimental observations (Paige et al., 1998; Viirre et al., 1986). Note that the pure vergence response seen in Figure 5 is combined with vergence modulations during each fast phase of nystagmus in Figure 6: this has been seen by Sylvestre et al. (2002) during visual disjunctive saccades, an emerging property.

We also tested the effect of head rotation frequency on the gain of the VOR while fixating central flashed targets at different depths. According to observations by Paige et al. (1998), the AVOR gain increases with rotation frequencies while fixating an *imaginary* earth fixed target in darkness. Moreover, in plots of the resulting VOR against concurrent vergence, they report that both the slope, and the intercept of this graph at 0 vergence, increase with rotation frequency (see Figure 8 in Paige et al., 1998). We emphasize the context of imaginary target, since our model does not include vision related loops in the light or constant visual input, but rather a vergence cue given by a flashed target.

**Figure 7:**
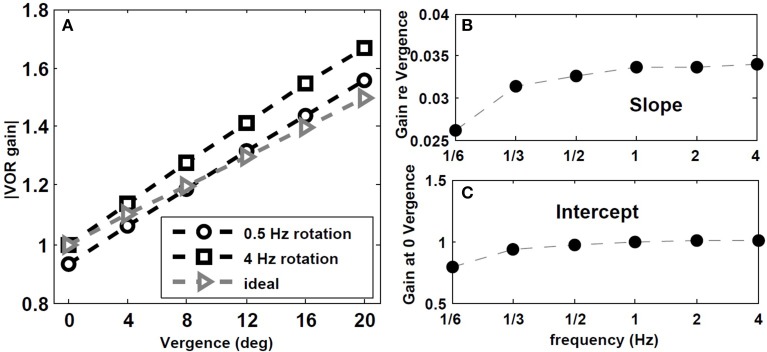
**Effect of rotation frequency on AVOR model gain. (A)** Absolute AVOR gain as a function of vergence during 0.5 Hz and 4 Hz rotation vs. ideal gains obtained from geometrical equations. **(B,C)** slope and intercept (at 0 vergence) of the AVOR gain vs. vergence as a function of rotation frequency.

To replicate this experiment, the hybrid model with *T_conj_* = 1.2 s is simulated with sinusoidal head rotation in the range of 1/6–4 Hz, with 30 degree/s peak head velocity while fixating central flashed targets at one of these distances: *D* = [2000 85.9 42.9 28.54 21.34 17.01] cm. Given the interocular distance and the rotation radius, the vergence angles for these target distances are: [ 0 4 8 12 16 20] degree, respectively. Figure 7 describes the effects of rotation frequency. The AVOR gain during low frequency rotation, 0.5 Hz, is closer to the ideal gains obtained from geometrical relations (for detail see Ranjbaran and Galiana, 2013a) compared to higher frequency rotation at 4 Hz (Figure 7A). Moreover, the slope and intercept plots (Figures 7B,C) show an increase with increasing frequency. These emerging results are in agreement with experimental observations of Paige et al. (1998).

### 3.3. Do changes in VOR anticipate changes in vergence angle?

The work of Snyder and King (1992) investigated the contribution of the vergence angle to VOR performance. In their experiments, they measured eye velocity in rotating monkeys while the vergence angle was required to change by flashing targets at different distances. Their results demonstrated that the VOR gain changed toward its correct value for the new target distance, before the correct vergence was acquired. They concluded that VOR gain modulation by target distance anticipates changes in vergence angle; thus, the binocular vergence angle alone, derived either from proprioception or from efference copy of motor command, is not sufficient to drive VOR modulation. They suggested that the transient discharge of gaze velocity Purkinje cells in the flocculus, associated with changes in vergence angle early enough, could drive VOR gain modulation with target distance (Snyder and King, 1992).

**Figure 8:**
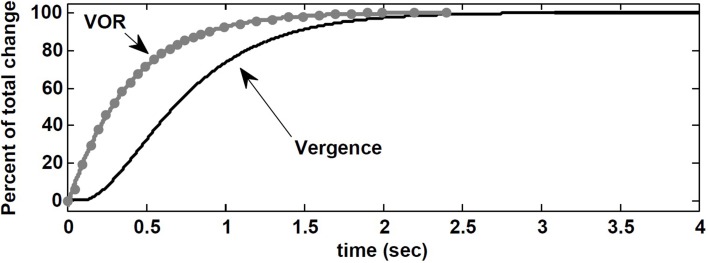
**Normalized vergence angle as a function of time for 8 degree vergence movement**. The flashed vergence stimulus occurs at time 100 ms. Gray dots (fitted by gray line) are normalized peak eye velocity resulting from brief head bumps at various instances during the vergence response. See text.

Here, we replicate their experiment with our nonlinear model. The visuomotor inputs, *Ve_R,L_* are applied after *t* = 100 ms such that a vergence movement is generated from 0 degree to 8 degree. This emulates a subject initially fixating on a far target straight ahead, and then fixating on a near central target at *D* = 43 cm. During this vergence movement lasting ≈ 2.5 s in our model, a vestibular input, i.e., a pulse of head velocity (accelerating with 500 degree/s^2^ to 30 degree/s, maintained for 40 ms and then decelerated) is added to the model and the peak eye velocity response is measured. As done by Snyder and King (1992), this vestibular input is applied at different times from 0 to 2.5 s in steps of 50 ms or 100 ms during the time course of the vergence movement (one pulse in each trace). Given the brief and small head perturbation, no fast phase is triggered. Figure 8 shows that the velocity of the eye movement evoked by VOR changed smoothly over the course of the 8 degree convergence. Both VOR peak eye velocity and vergence angle were normalized and replotted as functions of time to resemble the experimental results obtained by Snyder and King (1992) (their Figure 3). Our model simulations replicate their observations: VOR gain changes lead the vergence angle changes, and the change in VOR was completed before the vergence angle reached its goal.

Contrary to the suggestion by Snyder and King (1992), here, this observation is a result of VOR gain modulation by the *efference copies* of the vergence and monocular angles at the premotor level. In our nonlinear model, the effect of the required change in vergence appears in the efference copies *Ê*_*R,L*_ before *E_R,L_* via visuomotor projections to the PH. Consequently, the projections to the EHVs from the PH carry early vergence information to modulate the VOR gain, before the vergence movement actually begins or completes. It may appear that VOR changes anticipate vergence angle changes, but instead we suggest that the efference copies of the vergence angle modulating the VOR gain carry the vergence command before vergence responses appear at the behavioral level.

### 3.4. Response to steps in head velocity

Raphan et al. (1979) explored extensively the characteristics of VOR nystagmus during and after steps of passive head velocity, both in the dark and in the light. Eye velocity profiles in man and monkey decay to zero in the dark if the rotation interval exceeds 2–3 times the time constant of the canals. This is expected from a high-pass sensor. The simulations below focus on nystagmus in the dark, the context of our current model, using steps of head velocity of variable amplitude and duration.

#### 3.4.1. Per and post rotatory nystagmus

**Figure 9:**
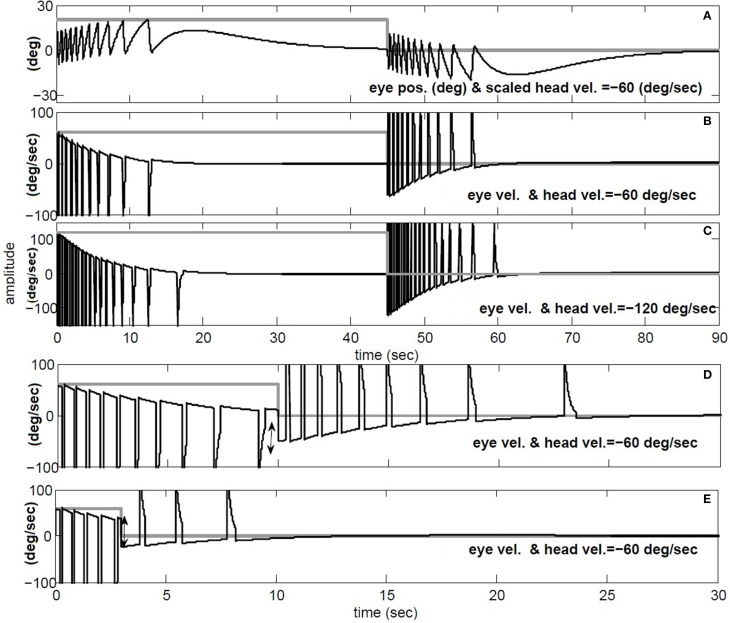
**Per and post rotatory nystagmus simulation in response to constant head velocity rotation. (A)** Conjugate position (degree) and **(B)** Conjugate vel. (degree/s) for for 45 s head rotation at −60 degree/s. **(C)** same as **B**, but doubling the speed of head rotation to −120 degree/s. **(D)** and **(E)** repeat head rotation velocity of −60 degree/s, now with short intervals of 10 and 3 s respectively. **(A)** black: conjugate position and gray: negative head vel./3. **(B–E)** black: conjugate vel., gray: negative head vel.

The simulations in Figure 9 replicate the main characteristics of VOR nystagmus during and after steps of head velocity. First, for long 45 s steps of rotation, it is clear that the post-rotatory nystagmus velocity appears equal in magnitude but opposite in direction to that during the rotation. In addition, the nystagmus velocity peak scales in both per- and post-rotation with the amplitude of the head velocity (Figures 9B,C). Second, for short duration rotations (Figures 9D,E), the initial post-rotatory nystagmus in the opposite direction is reduced in magnitude as the interval of rotation shortens. This is expected since the high-pass dynamics of the sensor will cause the *change* of nystagmus velocity to remain constant, if measured from the current eye velocity at the moment rotation ceases (see arrow in Figure 9D,E). As found in experimental data (Raphan et al., 1979), the frequency of fast phases in all cases is also modulated by the concurrent level of slow phase eye velocity.

#### 3.4.2. Dynamics of nystagmus decay

**Figure 10:**
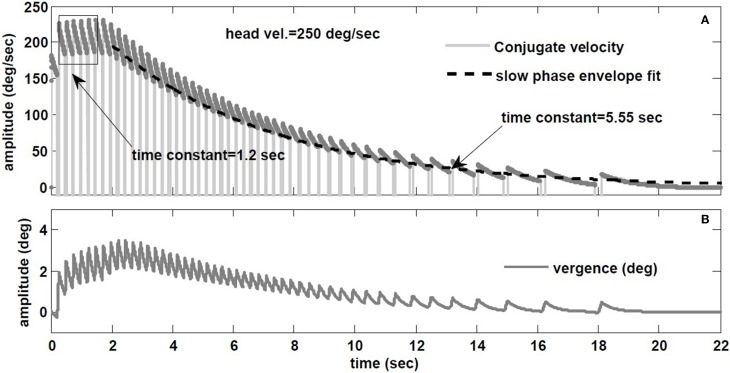
**(A)** Envelope fit on the decay rate of conjugate nystagmus velocity. **(B)** Vergence nystagmus associated with conjugate nystagmus in response to step rotation stimuli in the dark (prediction).

Raphan et al. (1979) also studied the decay rate of nystagmus velocity. As commonly done in the literature, they evaluated the dynamics of the VOR slow phase velocity by removing fast phases and replacing the gaps by interpolation: the reconstructed *envelope* was deemed to represent VOR dynamics, fitted with exponentials. The main result is that nystagmus decay appears much slower than the underlying slow-phase system (≈ 15 s vs. 4–6 s canal), hence the term *velocity storage* in the VOR coined by Raphan et al. However, an envelope fit ignores the contribution of initial conditions introduced at the start of each slow phase segment, biasing estimates of the slow phase time constant. To illustrate, Figure 10 provides the hybrid model response to a step of −250 degree/s in head velocity, with a canal time constant of 6 s and a conjugate slow-phase time constant of 1.2 s (see Table 1). The slow phase central time constant is intentionally low to highlight the effects, but these hold whenever there is nystagmus, especially at very low frequencies like steps. In Figure 10A, the *envelope* of slow phase velocities decays with a time constant of 5.55 s, despite slow phase central dynamics of 1.2 s. Such a response is often seen in unilateral vestibular patients. As discussed for sinusoidal rotations in Galiana (1991), ignoring the effect of nystagmus results in biasing the estimated conjugate VOR dynamics. There is a plateau-like response in the initial nystagmus velocity also seen by Raphan et al. (1979) at higher head speeds. Here it is caused by nonlinearities in the canal sensitivity, now exceeded by the input range. In addition, with the hybrid model, we predict the appearance of vergence nystagmus (Figure 10B) during step rotations in the dark. In order to extend *velocity storage* beyond both canal and central time constants, it is sufficient to incorporate the resting rates of sensors and central components (Galiana, 1991); at this time we only include modulations at all sites about resting rates, so only the central time constants can be *masked* during nystagmus.

## 4. Discussion

This paper introduces a hybrid nonlinear model to replicate human AVOR nystagmus in the dark. This bilateral model includes nonlinear sensors as well as nonlinear surfaces assigned to EHV cells to account for the target distance dependence of the VOR. It is shown that vergence can appear with both vestibular and visual depth stimuli. A physiologically relevant fast phase circuit and a nystagmus strategy are imbedded to generate nystagmus automatically and extend the functional range of the AVOR. In our former work (Ranjbaran and Galiana, 2013a), a comprehensive study was performed on the slow phase aspects of the VOR and their characteristics under different conditions. Here, we evaluated the performance of the hybrid model through simulations in response to passive head rotations with or without flashed visual goals. For the first time, a hybrid model based on known brainstem connections replicates different VOR characteristics, consistent with experimental observations. The comparison between the hybrid model simulation responses and experimental observations are qualitative at this stage, showing the capacity to explain reported behavior. Clearly experiments on individual subjects results in distinct numerical responses which require retuning of the parameters in the hybrid model.

Simulated nystagmus patterns replicate reported experimental observations (Figure 3) and trajectories that resemble human data (Figure 4). This suggests that the switching mechanism in our model is both plausible and testable with lesions and new inputs.

### 4.1. Disconjugacy of the AVOR:

Contrary to common belief, the AVOR is not purely conjugate in the dark; binocular recordings during sinusoidal rotations in darkness confirmed a vergence component in the AVOR (Khojasteh and Galiana, 2009a). In our model, this vergence component is a result of nonlinear sensors as well as nonlinear premotor cell responses that account for context dependent VOR responses. This suggests that local nonlinearities in the VOR circuit are the underlying mechanism for the disconjugate VOR in the dark.

### 4.2. Vestibular vergence interactions:

In addition to the vestibular input, i.e., head movement, visuomotor commands are included to enable vergence movements in response to *flashed* targets in the dark. Simulations show the effect of vergence goals during sinusoidal rotations: they confirm that the context dependency of the AVOR gain in the model is preserved with nystagmus and variable vergence goals. AVOR gain dependency on rotation frequency is also in agreement with experimental observations (Paige et al., 1998), an emerging property.

It appears experimentally that AVOR gain modulation with target distance precedes changes in vergence (Snyder and King, 1992), which questions vergence itself as the drive for AVOR gain modulation. We replicated this experiment (Snyder and King, 1992) with our model and found the same result: AVOR gain changes anticipate or precede the vergence profile (Figure 8). We conclude that AVOR gain modulation using efference copies of the vergence angle can support this anticipatory VOR modulation, even in the dark: we postulate that efference copies with visuomotor inputs affect EHV cells immediately, and so modulate the AVOR gain before the behavioral vergence is fully executed.

### 4.3. AVOR dynamics during steps of head velocity:

Per and post rotatory nystagmus characteristics are influenced by the peak velocity and duration of the stimuli. The proposed model replicates these data patterns (Figure 9). Given the switching aspect of nystagmus, we demonstrate that *envelope* measures provide biased estimates of slow-phase dynamics (Figure 10). So an important goal is to develop algorithms that provide unbiased estimates of nystagmus dynamics.

### 4.4. Testable predictions:

The goal of modeling a sensory-motor system is revealing potential strategies in the brain to control motion and to gain insight for clinical applications. Since the modeling results are fully consistent with available experimental data, the model structure warrants further study. Several assumptions or predictions remain to be verified: (i) Assumptions regarding anatomy of the VOR:

- Projections from brain centers (e.g., SC) to PH cells, carrying vergence *goal* information. These are necessary to cause VOR gain changes that precede the intended vergence change.
- Signals carrying on-going vergence angle information to EHV cells directly or via other VN cells to support target-distance dependent gain modulation of the VOR.
- The presence of premotor (e.g., PVP) projections to OPN cells, to enable the proposed switching strategy.

(ii) Predictions regarding VOR dynamics and behavior:

- Expected different monocular dynamics during fast phases directed temporally or medially (Equations 3A,B); This property could help distinguish between lesions in the burst circuits and those in the vestibular system.
- Increased vergence response after unilateral vestibular lesions; in addition to a decreased conjugate gain, unbalanced sensory projections in our bilateral model also predict an increase in the vergence response that is directionally assymetric relative to rotation direction, compared to a normal case.
- Biased estimation of the VOR dynamics using *envelope* approaches; the dynamics of AVOR slow phases should be estimated taking into account the effects of nystagmus and initial conditions.

In summary, we explored AVOR online gain modulation with target distance by introducing a physiologically relevant hybrid nonlinear model. We proposed local nonlinear computations at VN levels to account for the gain modulation of the VOR with context. It is likely that this hypothesis could also support long term adaptation or lesion compensation in the VOR. Furthermore, this hybrid model, given the realistic aspect of its simulated data, can also be used to generate virtual data for validation of algorithms that classify nystagmus segments and identify reflex dynamics.

## Funding

This work has been supported by Canadian Institutes of Health Research (CIHR), Natural Sciences and Engineering Research Council of Canada (NSERC) and Fonds de recherche du QuÉbec (FQRNT).

### Conflict of interest statement

The authors declare that the research was conducted in the absence of any commercial or financial relationships that could be construed as a potential conflict of interest.

## Appendix

### 5.1. Nonlinear surface

The nonlinear surface assigned to the EHV cells, *g_R,L_*, is obtained as a 3th order polynomial of *x* = *Ê*_*R*_ for the right EHV and *x* = *Ê*_*L*_ for the left EHV and a 1st order polynomial of *y* = *Ê*_*verg*_ according to the following equation:

(A1) $$g_{R,L}=m_{0}+m_{1}x+m_{2}y+m_{3}x^{2}+m_{4}xy+m_{5}x^{3}+m_{6}x^{2}y$$

For the model with *T_conj_* = 1.2 s, the coefficients in Equation (A1) are: *m*_0_ = 2.59, *m*_1_ = −8.051*e* − 5, *m*_2_ = 0.12, *m*_3_ = −4.8*e* − 6, *m*_4_ = 1.52*e* − 5, *m*_5_ = −4.12*e* − 6, *m*_6_ = −1.19*e* − 7.

For the model with *T_conj_* = 5 s, these coefficients are re-tuned to: *m*_0_ = 1.68, *m*_1_ = −6.43*e* − 5, *m*_2_ = 0.09, *m*_3_ = −3.84*e* − 6, *m*_4_ = −1.21*e* − 5, *m*_5_ = −3.29*e* − 6, *m*_6_ = 9.54*e* − 8.

### 5.2. Fast phase model equations

In this section, the equations to obtain the fast phase dynamic relations in Equations (3A,B) are described. Subscripts *R* and *L* refer to the right and left side of the brainstem, respectively. PVP, EHV, BDN, EBN, and IBN here refer to the net output from these cell populations. The parameters *a*, *p*_1_, *p*_2_, *d*, *m*, *b_I_*, *b_E_*, and α define weight of the projections between cell types or brainstem centers according to Figure 2C. Lower case letter *s* is the complex Laplace variable.

During a rightward fast phase, ipsilateral *PVP*s and *EHV*s and contralateral *IBN*s and *EBN*s are silenced. Signals from *BDN_L_* = *m* × *V_R_* − α × *Ê*_*R*_ are projected to *EBN_R_* and *IBN_R_*; therefore

(A2) $$\{\begin{array}{l} EBN_{R}=mV_{R}-\alpha {\hat{E}}_{R}\;\;\;\;EBN_{L}=0\\ IBN_{R}=mV_{R}-\alpha {\hat{E}}_{R}\;\;\;\;\;IBN_{L}=0 \end{array}$$

PVPs receive projections through linear summation of the ipsilateral canal *V*, contralateral PVPs and the PH. EHVs receive ipsilateral canal projections *V* as well as efference copies of ipsilateral eye position and vergence angle. The net output of EHVs, however, is scaled by a nonlinear gain that modulates the weight of canal projections according to the concurrent ocular angles (Ranjbaran and Galiana, 2013a); therefore,

(A3) $$\{\begin{array}{l} PVP_{L}=d{\hat{E}}_{R}+p_{1}V_{L}\;\;\;\;PVP_{R}=0\\ EHV_{L}=g_{L}p_{2}V_{L}\;\;\;\;\;\;\;\;EHV_{R}=0 \end{array}$$

Signals from VN and BN are added at MNs and then relayed to the eye plant $P_{f}(s)=\frac{k_{pf}}{Ts+1}$ to generate ocular movements:

(A4) $$\{\begin{array}{l} E_{R}=(-aPVP_{L}+b_{E}EBN_{R})\left(\frac{k_{pf}}{Ts+1}\right)\\ E_{L}=(EHV_{L}-b_{I}IBN_{R})\left(\frac{k_{pf}}{Ts+1}\right) \end{array}$$

Eye position efference copies *Ê*_*R,L*_ are available through projections from PH with similar dynamics as the eye plant, $F_{f}(s)=\frac{k_{ff}}{Ts+1}$; therefore,

(A5) $${\hat{E}}_{R,L}=\frac{k_{ff}}{k_{pf}}E_{R,L}$$

By substituting Equation (A5) into Equations (A2) and (A3) and combining with Equation (A4), one can obtain the dynamic equations provided in Equations (3A,B), to describe *E_R,L_*.
